# Chromosome-scale assembly of wild barley accession “OUH602”

**DOI:** 10.1093/g3journal/jkab244

**Published:** 2021-07-13

**Authors:** Kazuhiro Sato, Martin Mascher, Axel Himmelbach, Georg Haberer, Manuel Spannagl, Nils Stein

**Affiliations:** 1 Institute of Plant Science and Resources, Okayama University, Kurashiki 710-0046, Japan; 2 Leibniz Institute of Plant Genetics and Crop Plant Research (IPK) Gatersleben, Seeland 06466, Germany; 3 German Centre for Integrative Biodiversity Research (iDiv) Halle-Jena-Leipzig, Leipzig 04103, Germany; 4 Plant Genome and Systems Biology (PGSB), Helmholtz Center Munich, German Research Center for Environmental Health, Neuherberg 85764, Germany; 5 Center for Integrated Breeding Research (CiBreed), Georg-August-University, Göttingen 37075, Germany

**Keywords:** genome assembly, *Hordeum vulgare* ssp. *spontaneum*, OUH602, pseudomolecules, wild barley

## Abstract

Barley (*Hordeum vulgare*) was domesticated from its wild ancestral form ca. 10,000 years ago in the Fertile Crescent and is widely cultivated throughout the world, except for in tropical areas. The genome size of both cultivated barley and its conspecific wild ancestor is approximately 5 Gb. High-quality chromosome-level assemblies of 19 cultivated and one wild barley genotype were recently established by pan-genome analysis. Here, we release another equivalent short-read assembly of the wild barley accession “OUH602.” A series of genetic and genomic resources were developed for this genotype in prior studies. Our assembly contains more than 4.4 Gb of sequence, with a scaffold N50 value of over 10 Mb. The haplotype shows high collinearity with the most recently updated barley reference genome, “Morex” V3, with some inversions. Gene projections based on “Morex” gene models revealed 46,807 protein-coding sequences and 43,375 protein-coding genes. Alignments to publicly available sequences of bacterial artificial chromosome (BAC) clones of “OUH602” confirm the high accuracy of the assembly. Since more loci of interest have been identified in “OUH602,” the release of this assembly, with detailed genomic information, should accelerate gene identification and the utilization of this key wild barley accession.

## Introduction

Barley (*Hordeum vulgare*) was domesticated ca. 10,000 years ago in the Fertile Crescent. This important crop is found in most temperate climates worldwide (Bothmer *et al.* 2003). Cultivated barley (*H. vulgare* ssp. *vulgare*) and its wild ancestral form (*H. vulgare* ssp. *spontaneum*) are included in the same species and exhibit no reproductive barriers.

The center of distribution for ssp. *spontaneum* lies in SW Asia, particularly the Middle East (Bothmer *et al.* 2003). The wild barley accession “OUH602,” which has entirely dark spikes, is categorized as var. *transcaspicum* Vavilov and is endemic to Central Asia.

“OUH602” has been used as a key genotype in genetic and genomic studies. This accession has historically been used as a representative ancestral wild barley for linkage analysis by trisomic series (Takahashi and Hayashi 1962) and as a tester in differential hosts to define pathotypes of the powdery mildew pathogen *Blumeria graminis* f. sp. *hordei* (Moseman *et al.* 1965). Yun *et al.* (2005) also identified quantitative trait loci for multiple disease resistance derived from a cross between the resistant “OUH602” and cv. “Harrington”. From the same cross combination, recombinant chromosome substitution lines were developed to map morphological and agronomic traits (Yun *et al.* 2006; Gyenis *et al.* 2007). “OUH602” has also been used to generate expressed sequence tags (ESTs) (Sato 2020; see also https://harvest.ucr.edu/) (last accessed 2021-07-20). Using these transcript sequences, a high-density genetic map was constructed from a cross between cv. “Haruna Nijo” and “OUH602” (Sato *et al.* 2009), and recombinant chromosome substitution lines (Sato *et al.* 2009) were developed. A BAC library of “OUH602” was also constructed to isolate genes for major traits, *e.g.*, rachis brittleness (Pourkheirandish *et al.* 2015) and seed dormancy (Sato *et al.* 2016).

Following the release of a high-quality hierarchical BAC-by-BAC genome assembly (Mascher 2017), several whole-genome shotgun assembly techniques have been developed, *e.g.*, the DeNovoMAGIC assembly pipeline (NRGene, Nes Ziona, Israel), TRITEX (Monat *et al.* 2019) and w2rap-contigger (Clavijo *et al.* 2017). Using these assembly methodologies, the global landscape of the barley genome was recently analyzed using 20 cultivated and wild accessions (pan-genome: Jayakodi *et al.* 2020) based on diversity analyses on genotyping-by-sequencing (GBS) data of 22,000 accessions from the German National Genebank (Milner *et al.* 2019). Although the recently published pan-genome study also included one wild barley accession from Israel (B1K-04-12), the diversity information for the primary gene pool of barley (*H.* *vulgare*) is not yet sufficiently captured due to underrepresentation of wild barley assemblies (Jayakodi *et al.* 2021).

Here, we applied the TRITEX pipeline to generate a short read-based chromosome-scale *de novo* genome assembly of “OUH602.” We aligned the assembly to the most recently updated barley reference assembly, “Morex” V3 (Mascher *et al.* 2021), and the assembly of the Israeli wild barley accession “B1K-04-12” (Jayakodi *et al.* 2020) to highlight genomic variation among the genotypes. We also aligned the assembly to published BAC sequences of “OUH602”, obtained by Sanger sequencing used for gene isolation, to further benchmark the quality of the assembly.

## Materials and methods

### DNA extraction, library construction, and sequencing

High-molecular-weight DNA was isolated from leaf material of barley (*H.* *vulgare*) seedlings (Dvorak *et al.* 1988) and size selected for molecule size 40 kb or higher. Then, 440-bp paired-end (PE) libraries were prepared using a Hyper Kapa Library Preparation Kit (Kapa Biosystems, MA, USA) with no PCR amplification step. The 8- to 10-kb mate-pair (MP) libraries were constructed with a Nextera Mate Pair Library Sample Prep Kit (Illumina, CA, USA), followed by a TruSeq DNA Sample Prep Kit. The 10X libraries were constructed using a Chromium Genome Library Kit & Gel Bead Kit v2 (10X Genomics, CA, USA). The 440-bp PE libraries were quantified by qPCR and sequenced in one lane of a NovaSeq 6000 system for 251 cycles from each end of the fragments using an SP flow cell. The 10X and MP libraries were quantified by qPCR and sequenced in one lane for 151 cycles from each end of the fragments on the NovaSeq 6000 system using a NovaSeq S4 Reagent Kit. Fastq files were demultiplexed adaptors were trimmed from the 3'-ends of the reads with default adaptor stringency 0.9 by bcl2fastq v2.20 Conversion Software (Illumina). All libraries were prepared and sequenced at the University of Illinois Roy J. Carver Biotechnology Center. *In situ* Hi-C libraries were prepared as described by Padmarasu (2019). Sequencing data generated from each of the libraries are listed in Supplementary Table S1. The Hi-C data were used to prepare chromosome-scale assemblies using the TRITEX pipeline (Monat *et al.* 2019). The TRITEX pipeline was also used for contig assembly and scaffolding with PE, MP, and 10X data (Supplementary Table S1). The source code of TRITEX is available from Bitbucket: https://bitbucket. org/tritexassembly/tritexassembly. bitbucket. io/src/master/ (last accessed 2021-07-20). A detailed description of the pipeline is available here: https://tritexassembly. bitbucket. io (last accessed 2021-07-20).

### Gene projection

To derive projected gene structures for “OUH602”, we employed informant gene models of “Morex”, “Barke”, and "HOR10350", which have been predicted from transcriptome data and protein homology information (Jayakodi *et al.* 2020) using a previously described annotation pipeline (Mascher *et al.* 2017). The projection was based on a stepwise procedure as described (Jayakodi *et al.* 2020). Briefly, BLATN and Exonerate alignments of the coding sequences (CDS) to the “OUH602” genome sequence were computed for each of the three barley sources. Matches were clustered by their genomic loci and the top-scoring match was selected using a stepwise integration approach. The parameters for the integration rules are based on the same criteria described for the barley pan-genomes, prioritizing orthologous matches, uniqueness, match score, and completeness. In addition to protein-coding genes, the reported gene set also comprises 3432 pseudogenes that cover high-scoring matches with no contiguous ORF. Orthologs to selected lines of the barley pan-genome project were identified employing reciprocal best BLAST hits between protein-coding genes. Tandemly repeated genes in “OUH602” were detected as connected components from a similarity graph as described previously (Walkowiak *et al.* 2020). A maximum distance of nine unrelated genes between two tandem copies was selected to derive a threshold that is independent of variable physical gene densities in the genome.

### Repeat and transcript annotation

The final assembly was analyzed for repetitive regions using RepeatMasker (version 4.0.9) (Smit *et al.* 2013–2015) with the TREP Repeat library (trep-db_complete_Rel-19) (Wicker *et al.* 2002), changing repetitive regions to lowercase letters (-xsmall parameter) [repeat library downloaded from: http://botserv2.uzh.ch/kelldata/trep-db/downloadFiles.html (last accessed 2021-07-20)]. The output of RepeatMasker was condensed using the perl script “one-code-to-find-them-all” (Bailly-Bechet *et al.* 2014) with the parameters– strict and– unknown.

### Data validation and quality control

BUSCO with the plant dataset (embryophyta_odb9) was used for data validation and quality control. For gene prediction, BUSCO uses Augustus (Version 3.3) (Stanke *et al.* 2004; König *et al.* 2016). For the gene-finding parameters in Augustus, we set species to wheat and ran BUSCO in genome mode (-m geno -sp wheat).

### Alignment to published BAC sequences

Published “OUH602” BAC clone sequences of brittle rachis genes *Btr1* and *Btr2* (Pourkheirandish *et al.* 2015) and the quantitative locus seed dormancy 1 gene *Qsd1* (Sato *et al.* 2016) were downloaded from NCBI. Each BAC clone sequence was aligned with pseudomolecule sequences of “OUH602” and “Morex” V3 using minimap2 version 2.17 with the asm5 preset (Li 2018).

## Results and discussion

### Genome assembly

**Figure 1:**
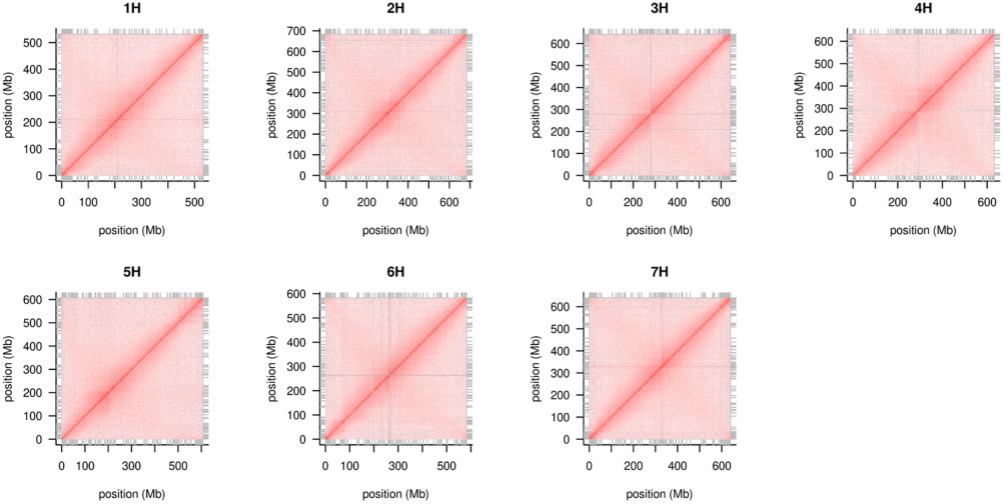
Intra-chromosomal Hi-C contact matrices. Gray lines mark contig boundaries. Some centromeres are spanned by a single scaffold. The absence of off-(anti)-diagonal signals supports the accuracy of the assembly.

**Figure 2:**
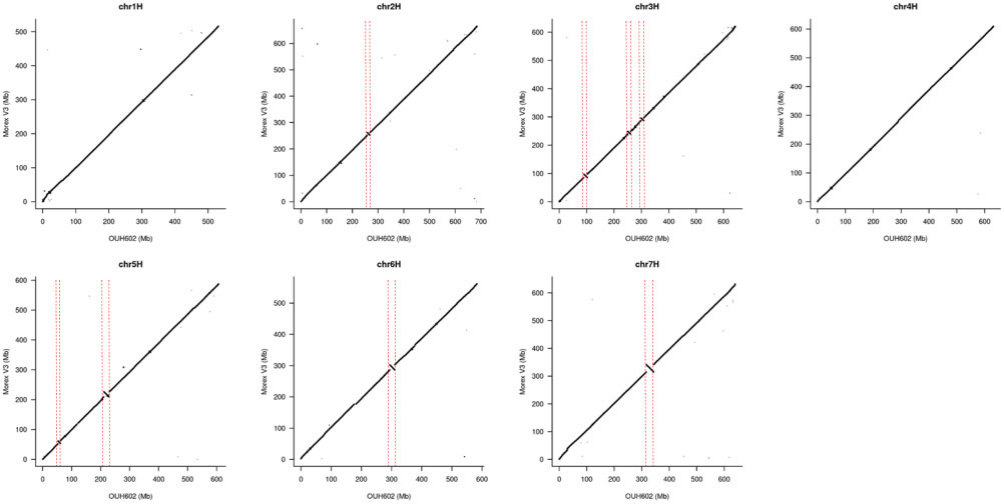
Alignment of pseudomolecules of “OUH602” to “Morex” V3 by a single chromosome. Areas between dotted red lines show inversions.

We generated the genome assembly from PE and MP short read and 10X Chromium linked read data. Approximately 813 Gb raw data provided an estimated 159-fold coverage of the genome (Supplementary Table S1). Assembly using the TRITEX pipeline (Monat *et al.* 2019) resulted in a scaffold N50 value of 11.3 Mb (Table 1). We integrated Hi-C data into the assembly, which uses native chromatin folding information to increase the contiguity to full chromosome size (Figure 1). The final pseudomolecule size was 4.32 Gb, comprising 736 scaffolds and a cumulative size of unanchored scaffolds of 177.5 Mb. The pseudomolecule size of “OUH602” is comparable with that in the pan-genome assemblies of wild barley accession “B1K-04-12” and cv. “Morex” V2 obtained using similar sequencing platforms but with a smaller scaffold N50 value. The quality of the “Morex” V3 assembly exceeded that of other assemblies due to the use of accurate circular consensus long-read sequencing on the PacBio platform (Mascher *et al.* 2021). The chromosome-wise alignment of pseudomolecules of “OUH602” to “Morex” V3 revealed conspicuous inversions on chromosomes 5H, 6H, and 7H (Figure 2), which is consistent with the results of Jayakodi *et al.* (2020), who reported that multi-megabase inversions are commonly found in barley. Apart from inverted regions, the overall contiguity of entire chromosomes was retained between “OUH602” and “Morex” V3.

**Table 1:** Statistics of “OUH602,” “B1K-04-12,” and two versions of “Morex” assemblies

Parameter	“OUH602”	**“B1K-04-12”** ^*b*^	“Morex” V2^*b*^	“Morex” V3^*c*^
Number of scaffolds in pseudomolecules	736	347	273	103
Pseudomolecule size (Gb)	4.32	4.27	4.34	4.20
Scaffold^*a*^ N50 [Mb]	11.3	35.5	43.7	118.9
Scaffold L50 [Mb]	116	34	27	12
Scaffold N90 [Mb]	2.1	5.5	5.9	21.8
Scaffold L90 [Mb]	433	142	122	40
Cumulative size of unanchored scaffolds (Mb)	177.5	56	82.9	29.1

a “Scaffold” refers to top-level entities that constitute the pseudomolecules. In “Morex” V3, these are Bionano scaffolds of PacBio HiFi contigs; in the other assemblies, these are superscaffolds constructed from PE, MP, and 10 X data.

b Data from Jayakodi *et al.* (2020).

c Data from Mascher *et al.* (2021).

### Quality of assemblies

We used the spectra-cn function from the Kmer Analysis Toolkit (KAT) (Mapleson *et al.* 2017) to check for content inclusion in the scaffolds and pseudomolecules. KAT generates a k-mer frequency distribution from PE and MP reads and identifies how many times k-mers from each part of the distribution appear in the assemblies being compared. It is assumed that with high coverage of PE reads, every part of the underlying genome has been sampled. Ideally, an assembly should contain all k-mers found in the reads (except k-mers arising from sequencing errors) and no k-mers that are not present in the reads (Schreiber *et al.* 2020). The spectra-cn plot in Supplementary Figure S1 generated from the contigs shows sequencing errors (k-mer multiplicity <20) in black, as these are not included in the assembly. Most of the content appears in a single red peak, indicating sequences that occur once in the assembly. The black region under the main peak is small, indicating that most of the content from the reads is present in the assembly. The content that appears to the right of the main peak and is present two or three times in the assembly represents repeated sequences. There are no obvious differences between pseudomolecules and scaffolds (Supplementary Figure S1).

We evaluated the quality of the “OUH602” assembly using BUSCO (Benchmarking Universal Single-Copy Orthologs, v3.0.2) (Simão *et al.* 2015; Waterhouse *et al.* 2018). This program assesses the completeness of a genome by identifying conserved single-copy, orthologous genes. The scaffold and pseudomolecule stages had complete single-copy genes at a rate of 95.4 and 95.5%, respectively (Table 2). These values are very close to those recently published for the “Morex” V2 assembly, with 97.2% single-copy genes (Schreiber *et al.* 2020). The differences are mainly due to the greater number of duplicated genes in scaffolds (1.7%) than pseudomolecules (1.2%). Only 0.9 and 1.0% fragmented sequences were present in scaffolds and pseudomolecules, respectively.

**Table 2:** BUSCO**^*a*^ statistics of “OUH602”**

Factor	Scaffolds	Pseudomolecules
Complete BUSCOs	1398 (97.1%)	1392 (96.7%)
Complete BUSCOs—Single-Copy	1374 (95.4%)	1375 (95.5%)
Complete BUSCOS—Duplicated	24 (1.7%)	17 (1.2%)
Fragmented BUSCOs	13 (0.9%)	14 (1.0%)
Missing BUSCOs	29 (2.0%)	34 (2.3%)
Total BUSCO groups searched	1,440	1,440

a Benchmarking Universal Single-Copy Orthologs, v3.0.2 (Simão *et al.* 2015; Waterhouse *et al.* 2018).

### Repeat masking

We analyzed each chromosome of the “OUH602” assembly for repetitive regions using RepeatMasker with the TREP repeat library. This analysis identified 72.0% (3.24 Gb) of the “OUH602” assembly as transposable elements (Supplementary Table S3), with the vast majority belonging to Class I (retrotransposons). The same analysis was performed for “Morex” V2 and “Morex” V3, producing similar results (Supplementary Table S3). The differences from the published results for the “Morex” V2 and “Morex” V3 assemblies (Monat *et al*. 2019; Mascher *et al.* 2021) are due to the different repeat libraries used.

### Gene projection

We assessed the gene content of “OUH602” using a gene projection approach as described by Jayakodi *et al.* (2020) for the 20 barley pan-genome assemblies. The total number of loci was 46,807, which is well within the range of 42,464–47,588 reported for the 20 pan-genome assemblies. Out of 43,375 protein-coding genes, between 42,800 and 43,211 loci exhibited a BLAST match with an e-value of <1–30, and 33,886 and 35,141 were one-to-one reciprocal blast orthologs between “OUH602” and “B1K-04-12” and “Morex” V2, respectively. Hence, the overall and orthologous gene content of “OUH602” is highly conserved in comparison to other barley lines. Likewise, 15.3% (6617) of the tandemly repeated genes in “OUH602” represented similar ranges as detected for the 20 barley pan-genome assemblies and were located in 2579 clusters. Thus, the gene content statistics above indicate that the “OUH602” assembly contains a gene set with highly similar characteristics to those reported for the 20 barley pan-genome assemblies.

### Alignment of BAC clone and pseudomolecule sequences

We aligned the pseudomolecule sequences of “OUH602” to pseudomolecules of wild barley accession “B1K-04-12” (Jayakodi *et al.* 2020) (Supplementary Figures S2 and S3). They both shared inversions on 5H, 6H, and 7H relative to the corresponding sequences in “Morex” V3 (Figure 2). The largest inversion between wild barley pseudomolecules was detected on chromosome 1H, which was not observed in the alignment between “OUH602” and “Morex” V3.

We aligned “OUH602” BAC clone sequences of three loci (*Qsd1* and *Btr1/Btr2*) to pseudomolecules of “OUH602” to estimate the contiguity of genome assembly (Supplementary Figure S4). These BAC clones were subjected to shotgun sequencing by Sanger sequencing and assembled on an individual clone basis.

The BAC sequences of *Btr1*/*Btr2* were composed of several clones and showed apparent discontinuity with the pseudomolecule sequence of “OUH602.” The alignment of these BAC sequences with the “Morex” V3 pseudomolecule sequence revealed fragmentation at the 3' region, but very high contiguity at the 5' region. Alignment of another BAC clone sequence, *Qsd1*, which was derived from a single clone, with pseudomolecules of “OUH602,” showed more contiguity; however, there was a significant gap between the BAC sequence and pseudomolecule sequence of “Morex” V3. The quality of the BAC sequences was comparable to that of “Morex” V3 but had some disorders that could be due to structural variation among genotypes. We should note that there are still mismatches between BAC clone sequences and pseudomolecules in “OUH602”; however, the per-base pair accuracy was high, with 0.36 and 0.04% divergence between pseudomolecules and BAC sequences at *Btr1/Btr2* and *Qsd1*, respectively, indicating that the pseudomolecules of “OUH602” are of sufficient quality to estimate the genic sequences on the genome.

## Conclusions

Here, we presented an assembly of the genome of a wild barley accession with nearly comparable quality to the recently published pan-genome assemblies. Because “OUH602” is often used as a genetic and genomic resource, this pseudomolecule sequence should add value to this resource and will promote further gene mining from this genotype. The “OUH602” assembly will help characterize additional sources of diversity to the primary gene pool of barley, which includes cultivated and wild ancestral forms of barley. The comparison between cultivated and wild barley assemblies may also promote the use of wild barley alleles in breeding programs.

## Data availability

Raw reads have been deposited in the ENA sequence read archive under Bioproject PRJEB44505 [Paired-end reads: ERS6294310, Mate-Pair reads: ERS6294311, 10X reads: ERS6294312, Hi-C reads: ERS6294314 and Assembly: ERS6294315] (Supplementary Table S2). The reference assembly is available for download or BLAST search from http://viewer.shigen.info/barley/index.php (last accessed 2021-07-20).

## Supplementary Material

jkab244_Supplementary_Data
