# Supplementary material for: Chromosome-scale assembly of wild barley accession “OUH602”
Source: G3 (Bethesda). 2021 Jul 13;11(10):jkab244. doi: 10.1093/g3journal/jkab244 (PMC8473966; doi:10.1093/g3journal/jkab244)
Supplement: jkab244_Supplementary_Data [file jkab244_supplementary_data.zip › jkab244-suppl_data/GENETICS-G3-2021-402537-s01.pptx]

## Slide 1
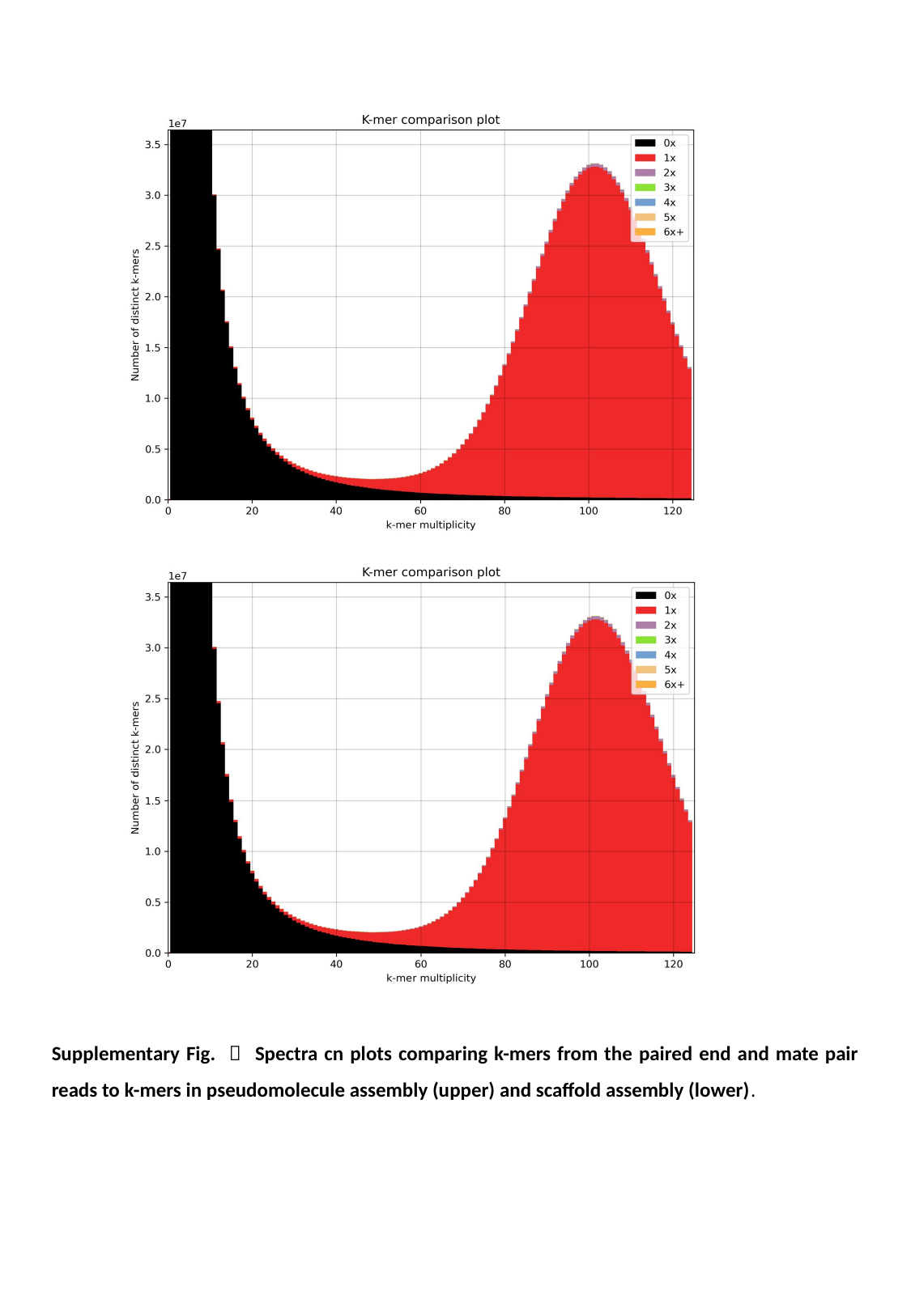

Supplementary Fig. １ Spectra cn plots comparing k-mers from the paired end and mate pair reads to k-mers in pseudomolecule assembly (upper) and scaffold assembly (lower).

## Slide 2
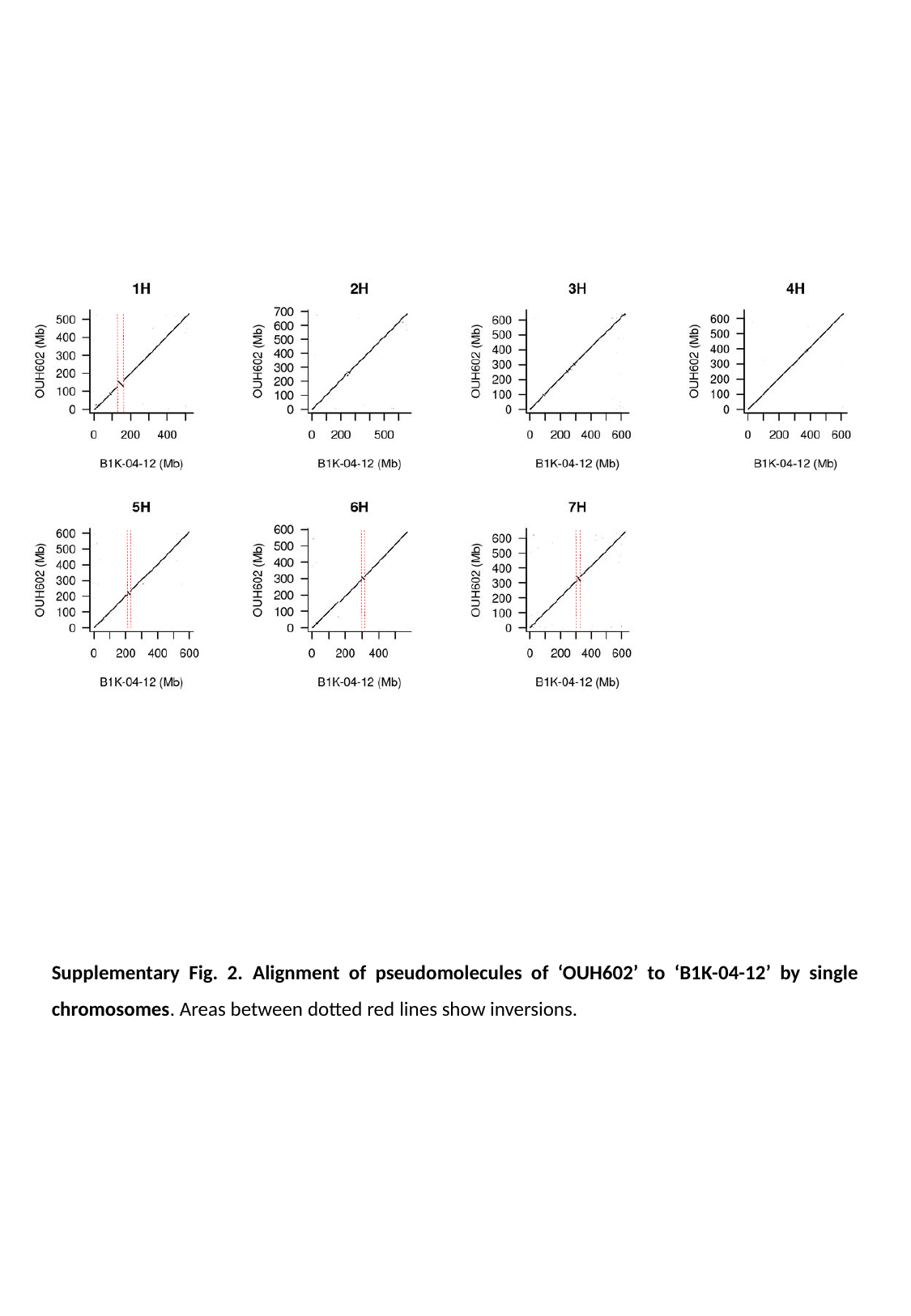

Supplementary Fig. 2. Alignment of pseudomolecules of ‘OUH602’ to ‘B1K-04-12’ by single chromosomes. Areas between dotted red lines show inversions.

## Slide 3
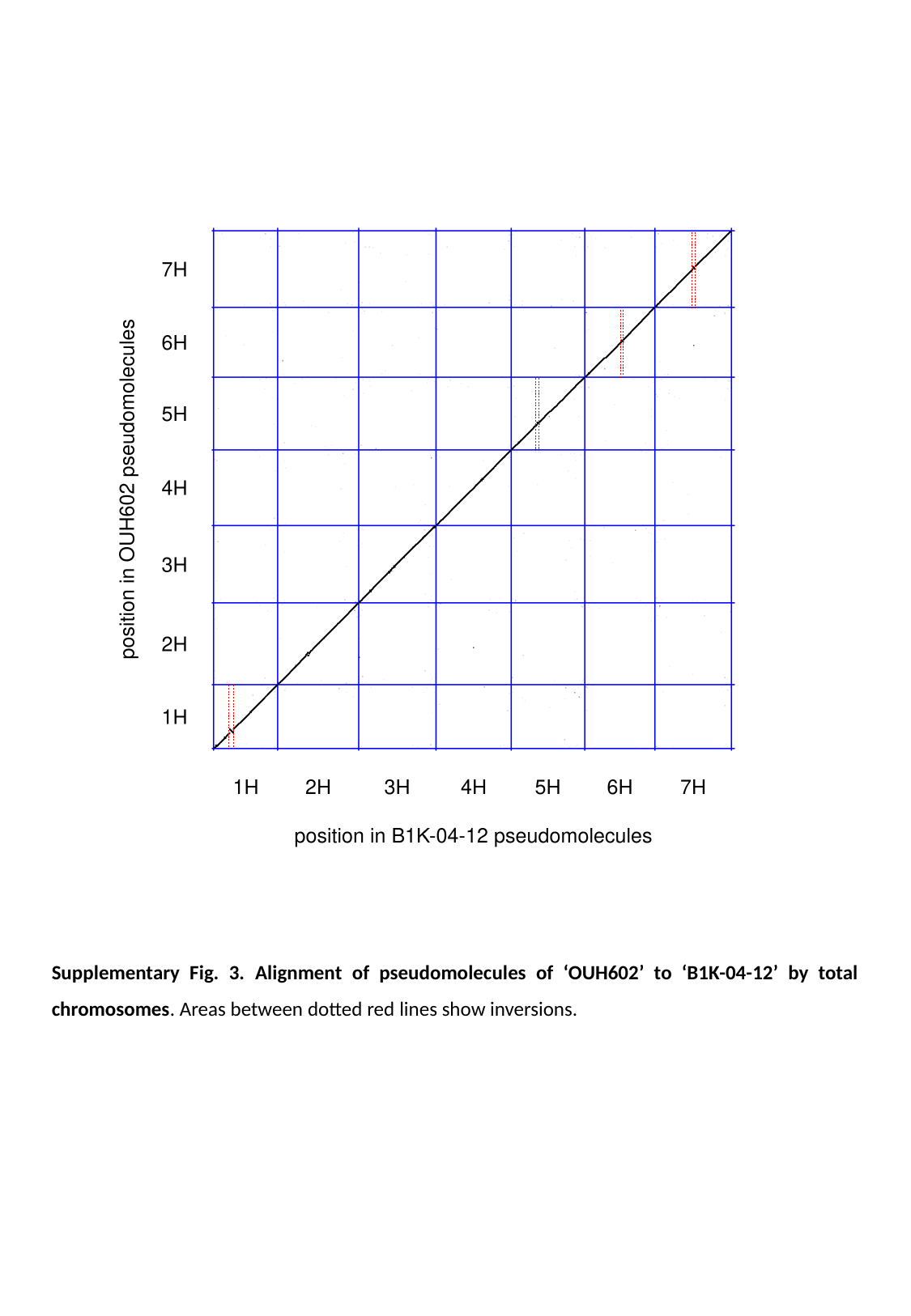

Supplementary Fig. 3. Alignment of pseudomolecules of ‘OUH602’ to ‘B1K-04-12’ by total chromosomes. Areas between dotted red lines show inversions.

## Slide 4
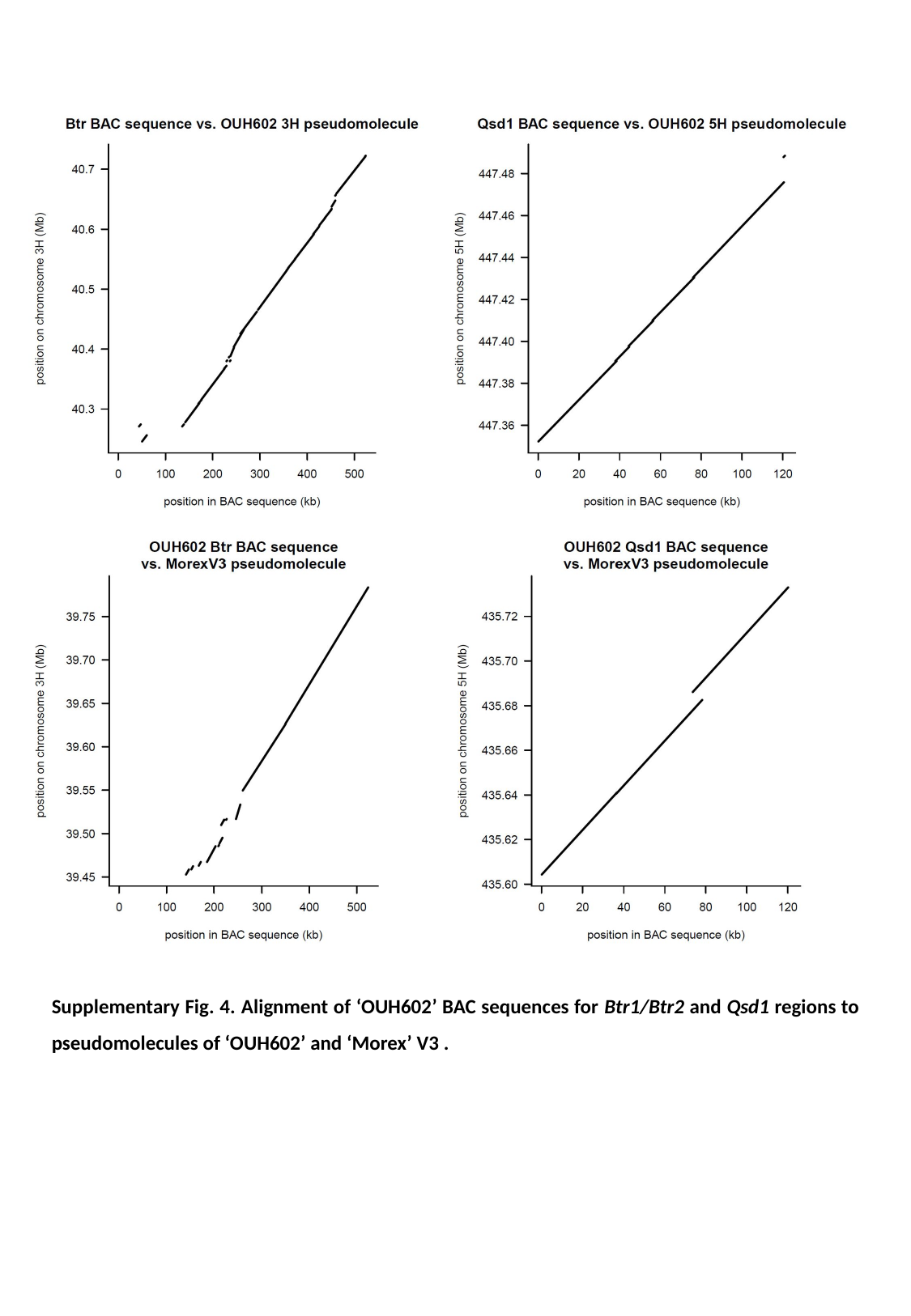

Supplementary Fig. 4. Alignment of ‘OUH602’ BAC sequences for Btr1/Btr2 and Qsd1 regions to pseudomolecules of ‘OUH602’ and ‘Morex’ V3 .
